# Manufacturing polymeric porous capsules

**DOI:** 10.1039/d1cc06565c

**Published:** 2022-03-17

**Authors:** Claudia Contini, Wenyi Hu, Yuval Elani

**Affiliations:** Department of Chemical Engineering, Imperial College London Exhibition Road London SW7 2AZ UK c.contini@imperial.ac.uk y.elani@imperial.ac.uk; FabriCELL, Molecular Sciences Research Hub, Imperial College London 82 Wood Lane London W12 0BZ UK

## Abstract

Polymeric porous capsules represent hugely promising systems that allow a size-selective through-shell material exchange with their surroundings. They have vast potential in applications ranging from drug delivery and chemical microreactors to artificial cell science and synthetic biology. Due to their porous core–shell structure, polymeric porous capsules possess an enhanced permeability that enables the exchange of small molecules while retaining larger compounds and macromolecules. The cross-capsule transfer of material is regulated by their pore size cut-off, which depends on the molecular composition and adopted fabrication method. This review outlines the main strategies for manufacturing polymeric porous capsules and provides some practical guidance for designing polymeric capsules with controlled pore size.

## Abstract 2

The review paper outlines the current state-of-the-art strategies adopted to fabricate polymeric porous capsules which represent a novel supramolecular construct that broadens the applicability and versatility of self-assembled polymeric systems.

## Graphical abstract

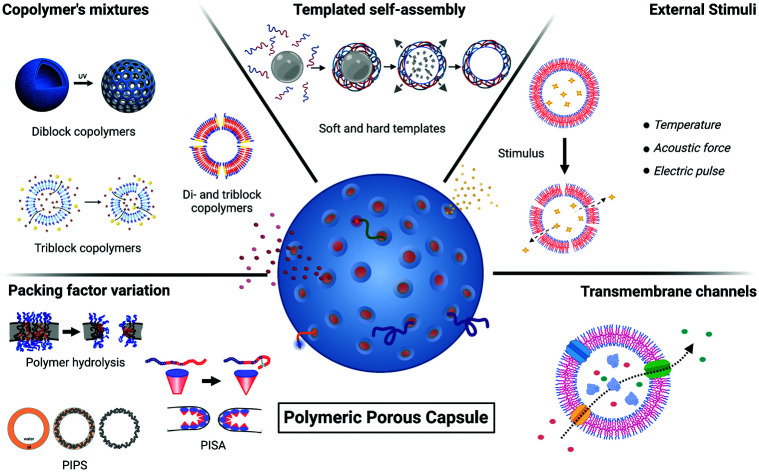

## 1. Introduction

Polymeric porous capsules have great potential in expanding the intercompartment communication capability of enclosed compartments with their environment. Part of their appeal is that they permit the isolation and protection of encapsulated cargo while controllably allowing the exchange of other materials. Inspired by biological compartmentalised systems,^1–3^ the ideal hollow capsules should have a well-defined and adjustable boundary to separate internal and external compartment volumes. This compartmentalisation could be accompanied by a tunable permeability and stable shell that allows an in and out exchange of defined molecules and an efficient retainment of its reactive macromolecular content while enabling, for example, the processing of biochemical reactions in its interior. Thanks to their enhanced and size-regulated permeability, polymeric porous capsules have applications in the fields of drug delivery,^4,5^ biosensing and bottom-up synthetic biology,^6^ for the engineering of a more sensitive through-shell communication, applied for gene expression,^7^ protein exchange^8^ and artificial quorum sensing.^6^

Molecular self-assembly is a formidable bottom-up approach used to produce functional and complex structures in nanotechnology and material sciences.^9–12^ Among the different materials and morphologies, polymeric capsules represent a versatile alternative to more conventional lipid-based structures (liposomes/vesicles), which are the basis of biological membranes and many therapeutic delivery systems.^13–15^ Compared with liposomes, polymeric capsules offer different intrinsic properties, including enhanced rigidity, chemical versatility, stability and mechanical properties.^14^ The use of synthetic polymers as opposed to lipids can also give access to an expanded chemical space, which can, in turn, be exploited for increased diversity of capsule properties and functions. Controlling their permeability through the introduction of pores is a powerful strategy that satisfies the requirements of various applications and allows enhanced control of their molecular exchange capabilities with the surrounding environment. Indeed, low permeability is a common shortcoming of existing lipid and polymeric self-assembled capsules, which often impedes their applicability in biotechnological and therapeutic areas.^15^ Porous structures have the potential to alleviate this limitation.

In cells, small and non-polar molecules can cross the membrane *via* simple (passive) Fickian diffusion. In contrast, the permeability of large and/or polar molecules (including ions) is achieved with the help of protein pores, channels, and carrier ionophores which enables more specialised processes of facilitated diffusion, or active transport. To mimic the environmental material exchange capabilities of living cells and create artificial nano and microreactors, the permeability of polymeric capsules can be controlled by producing pores with defined and tunable sizes, whose diameter depends on the capsule molecular composition and fabrication method. Pore size plays a dominant role in controlling the capsule shell's size-selective permeability: large pores can facilitate the influx and efflux of large macromolecules, while smaller pores may retain these, yet still allowing the exchange of small molecules. The optimal pore diameter depends on the dimensions of the molecule of interest, which could, for example, be a small molecule or nucleotide fragment.

Several detailed reviews exist about general polymersome physical chemistry, block copolymer synthesis, their properties, and their applications.^4,14,16,17^ Here, we focus specifically on porous polymeric capsules, with an emphasis on manufacturing methods. We aim to provide an overview of the different engineering strategies for porous polymeric capsules production to facilitate future research in this area, leaving the reader the choice of the optimal pore size depending on the molecular exchange/application that needs to be achieved. We will first outline some fundamental principles relating to polymeric capsules and then discuss the existing strategies for polymeric membrane poration. Broadly, five different approaches have emerged for engineering porous capsules, and we discuss each in turn: (i) packing factor variation of the polymeric building blocks; (ii) use of copolymer mixtures; (iii) templated self-assembly strategies; (iv) stimuli-responsive poration; (v) incorporation of biological nanopores.

### 1.1. Copolymeric self-assembly

**Fig. 1:**
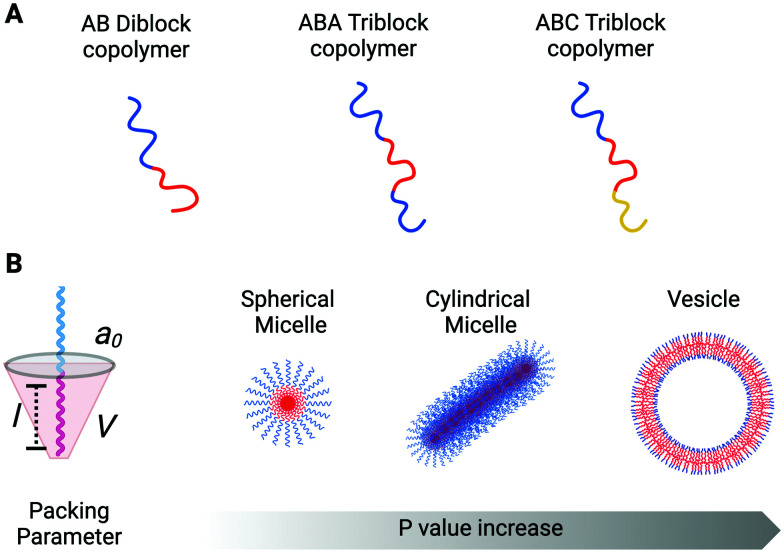
Schematic illustration of different copolymer and supramolecular structures at increasing packing parameter values. (A) Using diverse synthesis approaches, it is possible to obtain several copolymer architectures. (B) Three parameters (*a*_0_, *l*, *V*) define the packing parameter *p* of an amphiphilic copolymer (left). The hydrophilic block is represented in blue, while the hydrophobic is in red. Based on the *p* value, amphiphilic copolymer can self-assemble in spherical and cylindrical micelles and vesicles (right).

Copolymers are macromolecules composed of two or more blocks derived from more than one repetitive unit of monomers. Depending on the number of constituent polymeric blocks, it is possible to synthesise di-block, tri-block, tetra-block (and so on) copolymers. A general scheme for a di-block copolymer is A*n*–B*m*, where A and B are the two distinct constituent polymeric blocks, and *n* and *m* refer to the degree of polymerisation that belongs to each constituent block. In the same way, the general scheme for a tri-block, for example, can be either A–B–C or A–B–A (Fig. 1A). Hence, in a single block copolymer chain is possible to incorporate two or more polymeric units (blocks) with different physicochemical properties and abilities correlated to their structure. This is the case for amphiphilic block copolymers that incorporate their polymeric constituents' hydrophobic and hydrophilic characteristics within the same structure, facilitating self-assembly into organised aggregates of defined architectures.

The driving forces that lead to self-assembly are predominantly a combination of positive and negative interactions between hydrophilic and the hydrophobic regions (respectively) of the copolymer with the bulk aqueous environment. Free energy minimisation occurs when the hydrophobic polymer regions point towards each other, leading to the formation of thermodynamically more stable morphologies (*e.g.* spherical micelles and cylinders, spherical vesicles, lamellae structures).^18^

The morphology of the amphiphilic copolymer aggregates depends on the packing factor parameter, *p*, which predicts the curvature of the molecular assembly. The *p* value is correlated with the block copolymer geometry and properties, and it is defined by the ratio between three distinct factors: *p = V*/*a*_0_*l* (Fig. 1B). *V* is the volume occupied by the hydrophobic block, *a*_0_ is the optimal head-group area, and *l* is the length of the hydrophobic block in its fully straight configuration. Considering the packing factor, only amphiphilic block copolymers with a *p* value between 0.5 and 1 can arrange into bilayer membranes (including vesicles). All the rest will assemble into cylindrical (0.33 < *p* ≤ 0.5) and spherical (*p* ≤ 0.33) micelles or inverted colloids (*p* > 1).

**Fig. 2:**
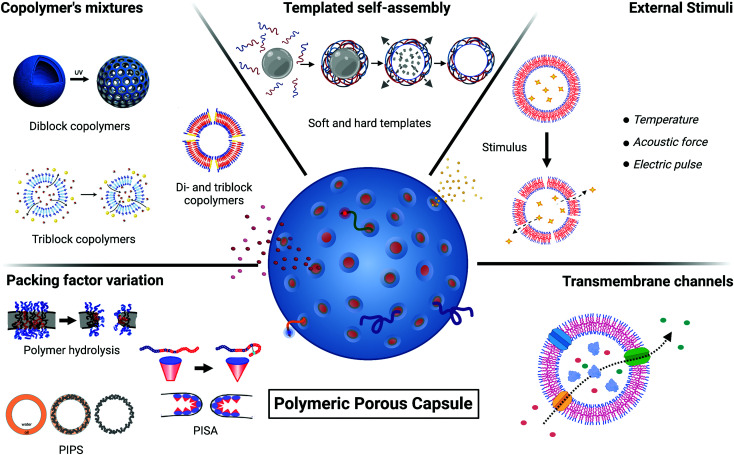
Schematic overview of different techniques for the generation of polymeric porous capsules. Polymeric porous capsules can be prepared using different strategies: variation of packing factor, copolymers’ mixtures, templated self-assembly, perturbating stimuli and insertion of transmembrane channels.

The bilayer formed by amphiphilic block copolymer is mechanically more resistant than lipid bilayers due to the entangled nature of the polymeric membrane, offering increased resistance to membrane disruption compared with their lipid counterparts. For this reason, the strategies of pore formation in polymeric vesicles need to overcome their mechanical stability to induce membrane deformations. In this review, instead of a comprehensive literature survey, we will use representative case studies as a basis for discussing the current state of the art strategies of polymeric membrane poration, summarised in Table 1 and Fig. 2. For some of the examples reported in Table 1, the detailed listing of capsule and pore dimensions is missing due to lack of sizes characterisation or high polydispersity.

**Table 1:** Summary table of the discussed pore-forming techniques. The table reports capsule and pore size range for each technique

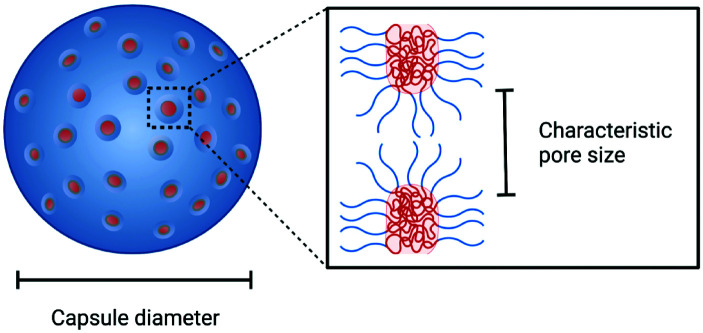			
Category	Method of pores formation	Capsule diameter	Pore dimension
Packing parameter variation	Polymer hydrolysis^20^	∼3 μm	5 nm
	Polymerisation-induced self-assembly (PISA)^28^	Micro-sized	Micro-sized
	Polymerisation-induced phase separation (PIPS)^38,39^	Micro-sized	200–300 nm ^35^ and from nano to micro size^36^

Use of copolymer mixtures	Di-block copolymers mixture^40^	∼150 nm	9–27 nm
	Tri-block copolymers mixture^41^	∼150 nm	MWCO between 50 to 1000 Da (∼1.1 to ∼2 nm)
	Di-block and tri-block copolymers mixture^43^	∼100 nm	∼5 nm

Templated self-assembly strategies	Soft templates^51^	2–10 μm	100–200 nm
	Soft templates – pickering emulsion^56^	∼1 μm	150 nm
	Soft templates – HIPE^46^	300–400	From nano to micro sized
	Solid templates^45^	100 nm	3.5–4 nm

Stimuli responsive poration	Temperature^47^	100–300 μm	∼10 μm
	Acoustic force^72^	Micro-sized	100–200 nm
	Electric pulse^75,76^	Micro-sized	Nano-sized

Transmembrane channels	Protein membrane^79^	Nano-sized	MWCO < 400 Da (1–2 nm)
	DNA nanopores^82^	∼100 nm	2 nm
	Artificial channels^83^	5–25 nm	Microsized

## 2. Packing parameter variation

The copolymer *p* value helps dictate the morphology and curvature of the assembled structures. An alteration of the *p* value provides a transition in the self-assembly capabilities of the copolymer towards other morphologies. This *p* value alteration can be used as a strategy to modify the general permeability properties of the polymeric assemblies and rationally design porous carriers. The alteration of the copolymer self-assembly capabilities and consequent poration is generated by a change in the local copolymer membrane bending and curvature. The generation of membrane pores is either induced by polymer degradation or during polymer synthesis.

### 2.1. Polymer hydrolysis

**Fig. 3:**
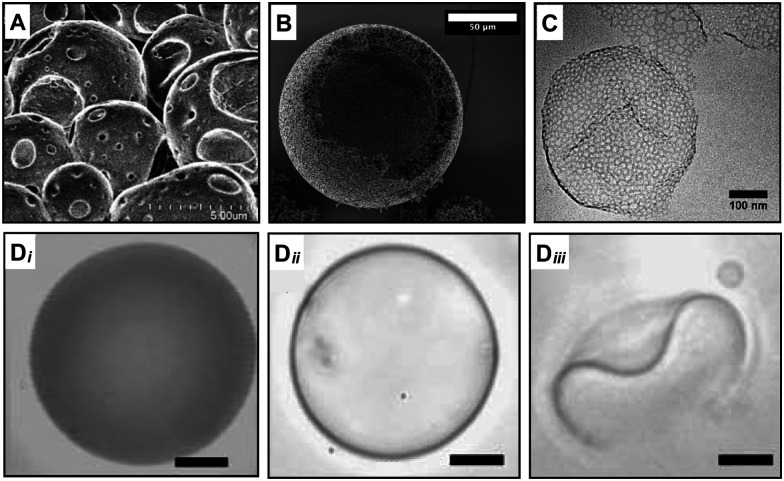
Microscopy characterisation of porous capsules made with a packing parameter variation and copolymers mixture strategy. The variation of the copolymer packing parameter leads to the generation of pores during (A) PISA^28^ and (B) PIPS.^38^ Scale bar = 50 μm. (C) Pores are also produced by mixing different curvature-forming copolymers in the same formulation.^40^ Scale bar = 100 nm. (Di–iii) The hydrolysis of one of the copolymers in the formulations leads to the formation of pores which causes an increase of the polymeric vesicle permeability (from Di to Dii) and a morphological change (Diii) over time.^20^ In this case, the nano-pores are below the resolution of the microscopy characterisation and pore size was inferred by other means. Scale bars = 5 μm. Figure adapted with permission from ref. 20,28,38 and 40 with permission from The Royal Society of Chemistry, copyright 2017, 2021 American Chemical Society and Elsevier respectively.

The key concept of this strategy is to use a mixture of block copolymers with one being the pore-generating component.^19,20^ In one example of this approach, polymersomes containing hydrolysable copolymers such as poly(l-lactic acid) (PEG-PLA) and polycaprolactone (PEG-PCL) were prepared in combination with inert polymer at various molar ratios to produce micro-sized porous capsules.^20^ The ester hydrolysis of PLA and PCL occurs in the hydrophobic block of the copolymer, causing an alteration of its volume and length and consequent shift of the packing parameter, *p* to lower values. The PLA or PCL polyesters hydrolysis in the core of the bilayer transformed these membrane-forming chains into detergent-like moieties. This transformation in copolymer properties generated high local curvatures in the bilayer, which drove the creation of hydrophilic pores and consequently triggered the release of encapsulants. The hydrolysis-triggered permeability was applied to control the release of encapsulants with different molecular weights, which determined a pore size cut-off of the shell of about 5 nm (Fig. 3D).

### 2.2. Polymerisation-induced self-assembly and phase separation (PISA/PIPS)

PISA and PIPS are “controlled” polymerisation techniques that allow the simultaneous synthesis of polymeric chains and formation of polymeric porous capsules (Fig. 3A and B). Controlled “living” polymerisations enable the fabrication of well-defined amphiphilic block copolymers from monomers to polymers. Ordinarily, the polymeric amphiphiles are first synthetised and then mixed in an aqueous solution to achieve self-assembled structures. In this polymerisation-induced self-assembly approach, the self-assembly of di-block copolymers is induced during the polymerisation of the second hydrophobic block, simultaneously achieving copolymer synthesis and self-assembly to nano-objects in one fabrication step.^21^ This approach reduces the number of steps required to produce polymeric vesicles.

#### 2.2.1. Polymerisation-induced self-assembly (PISA)

This approach uses the chain-end reactivity of solvophilic macromolecules (hydrophilic block) to polymerise a second monomer that becomes insoluble over a critical degree of polymerisation. The growth of the second block leads to the formation of complete block copolymers that self-assemble into nano-objects with different morphologies.^22^ PISA's most versatile and reliable polymerisation technique is the reversible addition–fragmentation chain transfer (RAFT) polymerisation technique, also known as “controlled” polymerisation. Apart from RAFT,^23–25^ many other controlled polymerisation techniques, such as nitroxide-mediated polymerisation (NMP),^26–28^ atom transfer radical polymerisation (ATRP),^19,29–31^ and ring-opening metathesis polymerisation (ROMP)^32–34^ have also been used in PISA to date.

PISA has become an effective method to produce porous capsules through the systematic variation of packing parameters and the ergodic morphology transitions, which depend on the choice of monomer and growth length of the solvophobic block.^21,35^ For example, Yoshida *et al.*^28^ prepared porous polymeric vesicles as an artificial model of a nuclear envelope. The perforated vesicles comprised of poly(methacrylic acid)-poly(methyl methacrylate-methacrylic acid-2,2,6,6-tetramethyl-4-piperidyl methacrylate), PMAA-P(MMA-MAA-TPMA) polymerised through photo NMP in an aqueous methanol solution. Due to the intramolecular interaction of acid–base groups (*i.e.* MAA and TPMA units), the hydrophobic chain changed its length causing a packing parameter change of the copolymer from a truncated cone to a cone-like shape. As the number of cone-like shapes increased at a much higher TPMA ratio, more rims were formed on the surface, leading to micro-sized pores on the membrane. Strong acids which disturb the MAA-TPMA interaction allows for changes in environmental pH to be used to control of the capsules' ability to retain and exchange cargo.^28^

Another study uses PISA to synthetise porous multipolymer vesicles which driving strategy of pore modulation is however through redox reactions of its ferrocene-containing triblock.^36^

#### 2.2.2. Polymerisation-induced phase separation (PIPS)

Another well-established procedure to prepare porous polymeric capsules involves polymerisation-induced phase separation (PIPS). PIPS is a method to separate reactive monomers from the initial homogeneous solution of a non-reactive component occurring during polymerisation.^37^ The PIPS of monomers in the oil phase of water-in-oil-in-water (w/o/w) double emulsions is followed by the removal of the inert diluent. The w/o/w droplets are usually prepared through microfluidics, and the PIPS process is initiated *via* UV light.^6,38,39^

Both Loiseau,^38^ Kim^39^ and co-authors prepared through microfluidics a mixture of UV-curable monomers (acrylate monomers), inert diluents (butanol and undecanol) and an initiator as the middle oil phase of the w/o/w double emulsions. During UV polymerisation, the monomers became insoluble in the diluent and started to phase-separate, yielding a homogenous porous polymer network characterised with 200–300 nm pores for Loiseau *et al.*^38^ and disperse porosity from nano to micro size for Kim *et al.*^39^ The degree of phase separation was adjusted by tuning the amount of inert diluent or the affinity of oil to monomers, which influences the microstructure and mechanical properties of the porous shell. This method allowed to achieve a selective and controlled release and uptake of molecules. Generally, higher diluent concentration and lower oil affinity to polymers resulted in a significant degree of phase separation, a larger porous network, and more fragile shells. Further, the fabricated porous microcapsules which encapsulated clay-DNA hydrogel were functionalised as cell mimics for achieving communication and quorum sensing in non-living mimics.^6^

## 3. Copolymer's mixtures

As the packing parameter dictates the polymer assembly curvatures and morphologies, it is possible to generate porous polymer vesicles by mixing different curvature-forming copolymers within the same formulation (Fig. 3C).

### 3.1. Diblock copolymers

Nanoporous polymer vesicles were produced using the amphiphilic polymer poly(ethylene glycol)-tetraphenylethene-cholesterol (PEG-TPE-Chol) whose hydrophobic block is constituted by the TPE part conjugated to a cholesterol moiety (Chol).^40^ The PEG-TPE-Chol exists in its two *cis*/*trans* stereoisomers, which have different self-assembly behaviours, with the *trans*- yielding vesicular structures and the *cis*-micelles. Mixtures of the two *trans*- and *cis*-isomers generated porous vesicles either *via* isomers mixing prior to vesicle formation (*trans*/*cis* 60/40 molar ratio) or *via* UV radiation of already performed *trans*-vesicles which generates a *trans*–*cis* photoisomeration. Both these strategies of *trans*-to-*cis* photoisomeration mixture produced pores of 9–27 nm in diameter.

### 3.2. Triblock copolymers

Polymeric film hydration followed by extrusion is a commonly used method to generate polymer vesicles. This method can furthermore yield porous shells when using specific block copolymers compositions. Schantz *et al.*^41^ have shown that the commercially available, nontoxic, low-cost triblock copolymers poly(ethylene glycol)-poly(propylene glycol)-poly(ethylene glycol) (also known as Pluronic® block copolymer) is commercially available example of structural material for the production of porous capsules. Mixtures of Pluronic® block copolymers such as Pluronic®-L121 (EO5-PO68-EO5, *M*_W_ ∼ 4400 g mol^−1^) and Pluronic®-F127 (EO100-PO65-EO100, *M*_W_ ∼ 12 600 g mol^−1^) produce stable, homogenous and permeable polymeric vesicles after hydration, sonication, incubation, freeze–thaw cycles and extrusion. A series mixture of Pluronic®-L121/F127 was initially prepared and analysed by Oh and colleagues.^42^ The authors found that the mixture of copolymers in a 1 : 1 mass ratio generated the most stable polymeric capsules. However, the permeability of the structure was explored only later by Schantz *et al.*^41^ The authors prepared the exact vesicle solutions using a sequential extrusion of polycarbonate filters of different pore sizes from 2 to 0.1 μm. By loading molecules of different molecular weights, Schantz *et al.* observed that the obtained capsules were porous and that the membrane's molecular weight cut-off was extrusion-tunable. The membrane's molecular weight cut-off was 50–400 Da and 600–1000 Da when the solutions were extruded using the smallest extrusion filters of 0.1 and 0.4 μm.

### 3.3. Triblock and diblock copolymers

Other copolymers such as poly(ethylene glycol)-polybutadiene (PEG-PBD) allow the assembly of polymersomes with increased stability but lower permeability. A novel direction is forming PEG-PBD vesicles with doped Pluronic® block copolymers, combining the merits of both di-block and triblock copolymers. Yan *et al.*^43^ mixed PEG(1300)-PBD(2500) and Pluronic®-P123 (EO_20_-PO_70_-EO_20_, *M*_W_ = ∼5 800 g mol^−1^) in four molar ratios and extruded them through 100 nm filters. The leakage assay indicated that the permeability of the membrane improved with the increase of the Pluronic®-P123 molar ratio. Vesicles with the 75 mol% of PEG-PBD and the 25 mol% of Pluronic®-P123 had an effective leakage of low-molecular-weight moieties (<5 kDa) and retention for high-molecular-weight substance (>10 kDa) with a pore size in the range of 5 nm. The authors have also demonstrated the biomedical application of these capsules as highly efficient magnetic resonance contrast agents.^43^ The same formulation has also been used to superoxide dismutase-loaded porous polymeric vesicles as an antioxidant formulation for neuropathic pain.^44^

## 4. Templated self-assembly

**Fig. 4:**
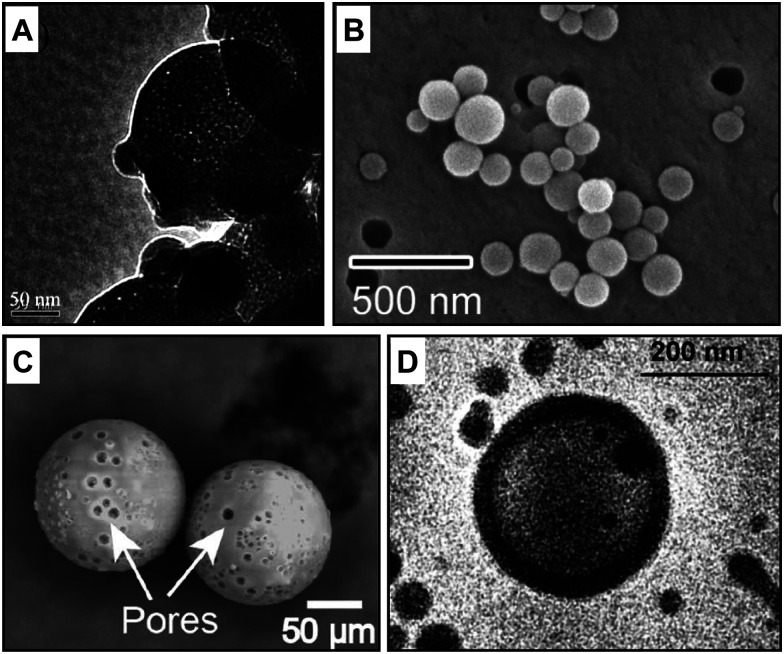
Electron microscopy characterisation of porous capsules made with templated self-assembly, stimuli-responsive and membrane channel insertion strategies. Examples of porous polymeric capsules made with (A) solid^45^ and (B) soft templates,^46^ (C) external stimuli^47^ and (D) protein channel insertion.^48^ Figure adapted with permission from ref. 45–48 Royal Society of Chemistry, copyright 2006 and 2019 American Chemical Society, 2020 Springer Nature and 2007 National Academy of Sciences, respectively.

Solid and soft templates have been applied to produce well-defined porous capsules whose morphology, diameter, and wall thickness are generally controlled by the material descriptors of the employed templates. Examples of soft and solid templated capsules are illustrated in Fig. 4A and B.

### 4.1. Soft templates

Colloidal soft particles, including vesicles and droplets, can be fabricated in a wide range of sizes from the nano to micro regime. They are convenient soft templates to yield well-defined capsules employing layer-by-layer (LBL) assembly of polyelectrolytes. The core–shell structure is constructed by depositing oppositely charged macromolecular species onto colloidal templates.^49,50^ The removal of the templating core by chemical or temperature decomposition allows the production of hollow capsules of different compositions with inherent permeability. For example, polymer capsules have been produced by LBL coating of positively charged FeII metallo-supramolecular coordination polyelectrolyte and negatively charged poly(styrene sulfonate) (PSS). These were templated onto a weakly cross-linked melamine formaldehyde (MF) core.^49^ The hollow and porous structure was obtained by subsequently decomposing the core with an acid solution of pH < 1.6.

By using a novel soft-templating approach, Amgoth *et al.*^51^ synthesised biocompatible nanoporous polymer capsules. A solution of polycaprolactone (PCL) was added dropwise and under continuous stirring into a MeO-PEG-NH-(l-GluA)_10_ solution, yielding PCL particles with MeO-PEG-NH-(l-GluA)_10_ spheroids inside and outside its structure. Further stirring was applied to dissolve the outer spheroids on the surface of the PCL structure. The outer spheroids dissolution led to the creation of pores of their equivalent size while the entrapped MeO-PEG-NH-(l-GluA)_10_ together with PCL-chain remained intact as a constituent of the capsules. Porous MeO-PEG-NH-(l-GluA)_10_-PCL capsules have been confirmed by FESEM, TEM, and AFM that the size of capsules ranges from 2 to 10 μm with an average pore size of 100–200 nm in diameter.^51^ As the building blocks are highly biocompatible, the nanoformulation was designed for therapeutic applications such as the controlled release of anticancer drugs (*e.g.* doxorubicin hydrochloride and imatinib mesylate).^51^

Pickering emulsions^52–55^ are a widely used soft template to produce porous microcapsules *via* the self-assembly of colloidal particles at the interface between two immiscible liquids.^56,57^ The stabilising colloidal particles form a flexible shell generated by locking colloidal particles together through thermal annealing, electrostatic binding, covalent cross-linking, van der Waals force, *etc.*^57,58^ with pores existing at the interstices between the stabilising colloidal particles. Dinsmore *et al.*^56^ emulsified water droplets in a solution of 90 vol% toluene and 10 vol% octanol containing carboxylate-modified polystyrene (PS) particles. Hollow capsules composed of 0.9 μm-diameter PS spheres and uniform 0.15 μm holes were produced when sintering the emulsion at 105 °C for 5 min. An increase in the sintering time led to smaller pores, and full particles coalesced after 20 min.

High internal phase emulsion (HIPE), a particular type of w/o/w double emulsion, is a concentrated system that possesses a large volume of the dispersed phase consisting of a volume fraction above 0.74.^59–61^ A unique class of porous materials, HIPE based polymeric (poly(HIPE)) materials, can be easily prepared in microfluidic devices. The monomers are contained in the water and/or oil phases. The generated poly(HIPE) materials are usually based on styrene, acrylates, and methacrylates and are used to produce various structures determined by the concentrated emulsion composition immediately prior to polymerisation. The hollow poly(HIPE) porous capsules were synthesised by creating HIPE droplets within a water phase in a microfluidic device, followed by the fast controlled Ostwald ripening of smaller droplets to produce larger droplets. The HIPE droplets are carried out by the flow and polymerised downstream *via* photopolymerisation.^62^

Like the templating w/o/w double emulsion mechanism, Dergunov's group^46^ produced porous capsules by loading polymeric building blocks, pore-forming templates, and cross-linkers into the hydrophobic interior of pre-formed vesicles made of lipid or surfactants. The polymerisation of the monomer generated a cross-linked shell with embedded pore-forming templates. After polymerising the monomers and removing the templates *via* solvent exchange, the obtained polymeric capsules possessed a controlled size distribution and tunable permeability. The approach enables control over thickness, shell stiffness and pore size uniformity by simply tuning the amount of polymerised monomer and using appropriate vesicle and pore-forming templates. Kim *et al.*^63^ also applied this vesicle-templating method to generate permeable polymeric capsules using butyl methacrylate, *t*-butyl methacrylate and ethylene glycol dimethacrylate monomers. Anionic (sodium dodecyl benzenesulfonate) and cationic surfactants (cetyltrimethylammonium *p*-toluenesulfonate) were used as a vesicle-templating scaffold.

### 4.2. Solid templates

Solid templates such as silica^64,65^ and calcium carbonate^66^ are commonly used to manufacture core–shell capsules by layer-by-layer (LBL) deposition of molecules. For instance, PCL nanocapsules were synthesised by depositing PCL chains onto the sacrificial silica nanoparticles with the help of a surfactant (*e.g.* Igepal CO-520). The surfactant helps the formation of micelles in between the deposited polymer.^45^ The removal of templating silica nanoparticles and surfactant micelles resulted in a hollow core and shell mesopores, with an average shell size of 100 nm and pore diameter of 3.5–4 nm, respectively. Besides the size of templates, depositing cycles and time also determined the shell wall thickness and pore size diameter.

Solid core/mesoporous shell (SC/MS) particles, prepared in various sizes and pore diameter, shell thickness and solid core composition (*e.g.* silica, gold, Fe_3_O_4_ nanoparticles), can also be applied as scaffolds for the assembly of macromolecules. Wang *et al.*^67^ used the SC/MS particle with a solid silica core (*ca.* 300 nm) and mesoporous silica shell (*ca.* 60 nm) to infiltrate and adsorb the solution of poly(allylamine hydrochloride) (PAH) molecules. This was followed by the covalent cross-linking of the infiltrated PAH chains in the mesoporous silica shells and subsequent removal of the SC/MS silica templates, resulting in monodisperse and single-component polymer capsules. The single assembly process is more efficient than multiple LBL deposition. Capsule thickness and physical properties are easier to tune simply through the construction of the various mesoporous shell and the cleavable covalent linker molecules.^67^

## 5. Stimuli-responsive techniques

Stimuli-responsive polymeric materials^11,68–70^ can adapt and respond to changes in the surrounding environment. External stimuli such as temperature, acoustic and mechanical deformation can generate and control the opening and closing of pores on capsule shells. It should be noted that, in most cases, the stimuli-generated pores are only transient and close as soon as the stimulus is terminated.

### 5.1. Temperature

Temperature is a widely-used trigger for the control of loading and release, particularly in biomedical contexts. Thermosensitive polymers exhibit reversible thermo-responsive phase transition at their lower critical solution temperature (LCST). Controlling the assembly and disassembly of block copolymers through temperature change can lead to the “on–off” switch of membrane permeability. Rahman and Elaissari^71^ prepared the polymeric capsule consisting of the thermo-responsive block copolymer poly(*N*-isopropyl acrylamide-aminoethyl methacrylate) (P(NIPAm-AEM)), by emulsion polymerisation, cross-linking and magnetic core removal. The thermosensitive polymer P(NIPAm-AEM) underwent volume phase transition above its LCST (around 50 °C), tuning the permeability of small molecules through open pores at *T* < LCST and close pores at *T* > LCST.^71^

Porous microcapsules with a diameter range of 100–300 μm and a shell thickness of 800 nm have been generated by combining thermally induced polymerisation with a microfluidic flow-focusing device (MFFD). Biphasic droplets comprising a silicone-oil core and acrylate-monomer shell were generated using an MFFD and served as a soft template to generate porous microcapsules *via* off-chip thermal polymerisation. Even if the pore size cut-off has not been investigated yet for these porous structures, a ∼10 μm pore size diameter is visible in the EM images (Fig. 4C).^47^

### 5.2. Acoustic forces

Gigahertz (GHz) acoustics can exert mechanical forces and deformation on the membrane, resulting in transient pores on the shell. Lu *et al.*^72^ used a nanoelectromechanical resonator to generate GHz acoustic streaming on polymer-shelled vesicles and produced transient pores which ranged from 100 to 200 nm in diameter. This is a non-invasive method to produce porous capsules with controlled loading and release of encapsulants by opening and closing pores. The rate of loading or release is determined by GHz acoustic streaming. The more significant release occurred when applying higher power for more extended times.^72^

### 5.3. Electric pulse

Use of electric pulses is another common approach to induce pores by generating transient and dynamic deformations. The degree of deformation depended linearly on both the magnitude of the applied electric field and the intra-to-extracellular conductivity ratio.^73,74^ Above the critical transmembrane potential, intense electric pulses induce electric breakdown, resulting in the nucleation and expansion of membrane defects. This leads to membrane permeability through the influx/efflux of molecules *via* transient pores across the bilayer. For example, Yun *et al.*^75^ and Yoshida *et al.*^76^ reported the control of composite capsules permeability and increase of molecular release rates in the presence of an electric field.

## 6. Insertion of transmembrane channels

Polymeric vesicles can be functionalised with transmembrane channels to achieve a size-selective permeability and exchange of ions or small molecules. This approach has been employed with lipid membranes,^77^ for example, using protein pores and blockers, which act as molecular regulators to tune permeability.^78^ Similar biomimetic strategies to form polymeric capsules are increasingly being explored.

### 6.1. Membrane proteins

Membrane proteins have been used to increase the permeation capabilities of polymeric capsules. In nature, channel proteins are embedded into lipid matrix, and they often require specific membrane composition and thickness to be fully functional. However, there are several examples in the literature where the hybridisation and reconstitution of channel proteins into polymeric matrix have been successful. Bacterial porin OmpF has been reconstituted into triblock copolymer vesicles (poly(2-methyloxazoline)-*block*-poly(dimethylsiloxane)-*block*-poly(2-methyloxazoline)) to allow a passive exchange of molecules with a molecular weight lower than 400 Da and the entrapment of proteins in the vesicles lumen. The porin can be incorporated either into pre-formed vesicles or during the vesicles’ preparation during solvent exchange.^79^ The strategic inclusion of a pH-responsive molecular cup in the outer part of the porin allows further regulation of the molecular flow across the membrane in a stimuli-responsive manner.^80^ This pore-vesicles system has been shown efficient as a polymeric nanoreactor for the local production and release of antibiotics due to the entrapment of biocatalysts in the lumen and permeation of substates. The same research group engineered porous polymeric vesicles by inserting other channel proteins (LamB^81^ and AqpZ^48^). An example of EM characterisation of inserted protein channel is in Fig. 4D.

### 6.2. DNA nanopores

Another route for controlling the transport across membranes is the engineering of DNA-based nanopores made through the controlled self-assembly of oligonucleotides. The control over design allows to tailor the pore diameter and regulate the transport across the membrane. Polymersomes composed of a polymethacrylate matrix have been functionalised with cholesterol-anchored DNA nanopores by simply incubating the pores with polymersomes. The polymeric capsules exhibited a size-dependent permeability compatible with the nanopore lumen diameter of 2 nm, where peptides transport across the membrane is enabled while large enzymes are retained.^82^

### 6.3. Artificial transmembrane channels

Artificial pH-responsive transmembrane channels can be installed in the polymeric capsule walls.^83^ Polymeric regions comprising of acrylic acid units formed the bilayer islets within the polymeric vesicle. The change of the external pH from 5 to higher values led to the opening of the channel which were accessible also to large cargos such as haemoglobin. The bilayer islets offered a channel size range from 5 to 25 nm.

## 7. Discussion and outlook

Each of the porous capsule manufacture strategies outlined above comes with its advantages and limitations which are summarised in Table 2.

**Table 2:** Summary table of the advantages and disadvantages of each engineering strategies. The table highlights advantages and disadvantages for each discussed technique

Category	Advantages	Disadvantages
Packing parameter variation	• Simple deployment	• Poor control over morphology and size distribution
	• Coupling capsule fabrication with pore formation	• Limited ability to form higher-order morphologies
	• Does not require bespoke instrumentation or extensive training	• Low encapsulation and cargo release efficiency

Copolymer's mixtures	• Fast kinetics of capsule formation	• Poor control of size distribution
	• Does not require bespoke instrumentation	• Low encapsulation and cargo release efficiency

Templated self-assembly	• Control over morphology and size distribution	• Slow kinetics of formation
	• Higher stability and controllability	• Use of surfactants
	• Allows layer-by-layer strategy	• Requires additional step of template removal

Stimuli-responsive	• Allows smart content-release strategies	• Poor control over pore size distribution
		• Transient pore formation

Transmembrane channels	• Increased design space by combining synthetic and biological components	• Requires optimisation in the insertion of the channels
	• Incorporation of biological function within a synthetic matrix	• The nature of the transmembrane channel typology limits pore size

Those methods that rely on changing the polymer composition of the capsule for porosity (*e.g.* by deploying copolymer mixtures or using a variation of packing parameters) have the advantage that they are simple to deploy. The production processes generally involve mixing, hydration, and agitation. As it relies on molecular self-assembly, and the methods themselves do not require extensive training, optimisation, and bespoke instrumentation. Despite the straightforward process, however, the use of aqueous solvent and mechanical agitation is often time-consuming and usually results in the production of capsules with poor control over size distribution and often low encapsulation and release efficiencies, compared with templated and microfluidics techniques.

Methods that rely on polymerisation-induced self-assembly and phase separation (PISA/PIPS), on the other hand, have the advantage of coupling an efficient fabrication process with capsules of increased stability and rigidity. Moreover, polymerisation-induced synthetic techniques make it a versatile method due to the tolerance to a broad range of reaction conditions and monomer families. However, the kinetics of high-ordered morphologies formation is greatly limited by the low chain mobility of the solvophobic blocks.

Techniques involving templating self-assembly yield more stable porous capsules. Moreover, the capsules tend to be more monodisperse, as the template scaffolds generally have a uniform size distribution. In addition, the geometry of porous capsules and pore size can be easily controlled by the employment of templates of a given shape and size. Nevertheless, the commonly used layer-by-layer loading process is time-consuming and limited by the functionality of surfactants.

Proration strategies that rely on stimuli are attractive due to their wide deployment in content-release applications. However, it is difficult to exert control over the size of the pores, and with some exceptions, pore induction tends to be transient (*i.e.* the pores seal up when the stimuli are removed).

Despite the increasing complexity of processing procedures, the combination of various fabrication techniques usually leads to better control of pore generation on polymeric capsules. For example, combining the PIPS with the double emulsion templates permitted more accurate control over capsule dimensions than conventional emulsification techniques.

Examples of different manufacturing approaches for generating porous capsules are proliferating rapidly, and several areas for future growth can already be identified based on existing trends. The first is the increased use of microfluidics^84,85^ to control the size and architecture of the assemblies. This is especially promising given the effect that ‘cleanroom free’ microfluidics has had in democratising the field, allowing researchers working in diverse subject areas to build their own devices using 3D printing and other rapid prototyping technologies.^86,87^ A second is a shift towards greater precision engineering of porous structures, for example, where the number and pores in the capsules can be precisely controlled.^85^ Finally, the manufacture of structures is expected across vastly different length scales. With lipid structures, extensive microfluidic solutions, in particular, have been employed to construct compartments at both the micro- and nano-regimes, and analogous platforms will likely be developed for the porous polymeric capsules.^88,89^ Now that generation procedures are becoming more robust, a third future growth area is a move towards applications. These have been limited to proof-of-concept studies for demonstrating their potential use as chassis for synthetic cells in bottom-up synthetic biology, in drug and gene delivery, and as microreactors in a number of landmark papers; future years will likely see an increased number of application-focused studies, as well as increasing deployment of porous capsules in real-world settings. Most experimental set ups are at the proof-of-concept level and, despite the proposed uses in many applications, the promise of such porous capsule is still not realised. Our hope is that this review will offer an overview guideline on the existing manufacturing methods of porous polymeric capsules and inspire their use for more concrete applications and uses.

## Conflicts of interest

The authors declare no conflict of interest.

## Supplementary Material
